# Endoscopic Coil Embolization for Refractory Intrahepatic Biliary Duct Leak

**DOI:** 10.14309/crj.0000000000000743

**Published:** 2022-02-23

**Authors:** Barbara Dutra, Macartney Welborn, Nirav C. Thosani, Ricardo Badillo, Tomas DaVee, Dimpal Bhakta

**Affiliations:** 1 Department of Internal Medicine, University of Texas Health Science Center at Houston, Houston, TX

## Abstract

Bile leaks may be seen after blunt and penetrating trauma, as well as iatrogenic injury from surgical procedures. There are many articles on endoscopic treatment options for the management of biliary leaks, including sphincterotomy, endoscopic stent, or nasobiliary drain placement. Data, however, are scarce regarding the management of persistent biliary leaks after the initial intervention. We present a case of endoscopic coil embolization to treat a refractory bile leak after initial endoscopic sphincterotomy and stent placement in a patient with a grade IV liver laceration due to a gunshot wound.

## INTRODUCTION

Biliary leaks are seen after abdominal trauma and surgical cholecystectomy.^1–3^ The American Society for Gastrointestinal Endoscopy has recommended sphincterotomy, endoscopic stent, or nasobiliary drain placement as options for the management of postoperative bile leaks.^4^ These modalities have been used as primary interventions for bile leaks from other etiologies.^5–8^ Although successful treatment of biliary leaks with endoscopic intervention has been reported to be as high as 80%–100%, there is no consensus on the preferred endoscopic modality for managing biliary leaks.^4^ Furthermore, data are scarce regarding the management of refractory biliary leaks. Given the risks associated with persistent bile leaks and lack of consensus in therapeutic choices, innovative endoscopic modalities have been pursued, including the use of endovascular coils for biliary leak embolization.^9–15^ We present a case of endoscopically placed metallic coils for embolization of a refractory bile leak after penetrating abdominal trauma.

## CASE REPORT

**Figure 1.**
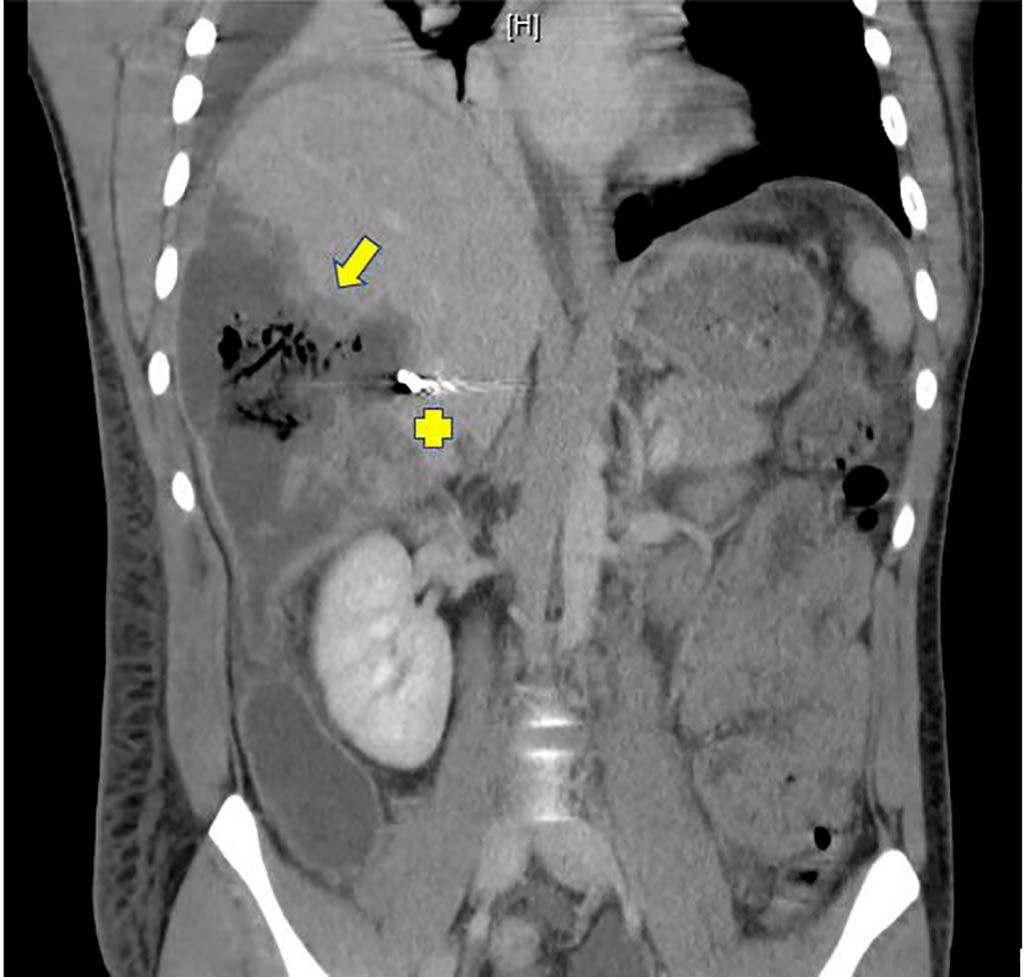
Complex right hepatic lobe necrotic collection measuring 13 by 8 cm with internal gas bubble formation (yellow arrow) with endovascular coils noted in branches of the hepatic artery (yellow cross).

A 21-year-old man without significant medical history presented after gunshot wounds to the right upper quadrant and right shoulder. On presentation, the patient was found to have radiographic evidence of grade IV liver laceration, pneumoperitoneum, right pneumothorax, and multiple rib fractures. His hospital course was complicated by persistent low-grade fevers, leukocytosis, and right upper quadrant pain. Imaging revealed a complex right hepatic lobe necrotic collection measuring 13 by 8 cm with internal gas bubble formation, for which interventional radiology placed a 22 French intrahepatic drain (Figure 1). Interventional gastroenterology was consulted because of concern for biliary leak on postoperative day 15 due to voluminous bile draining from the right intrahepatic drain.

**Figure 2.**
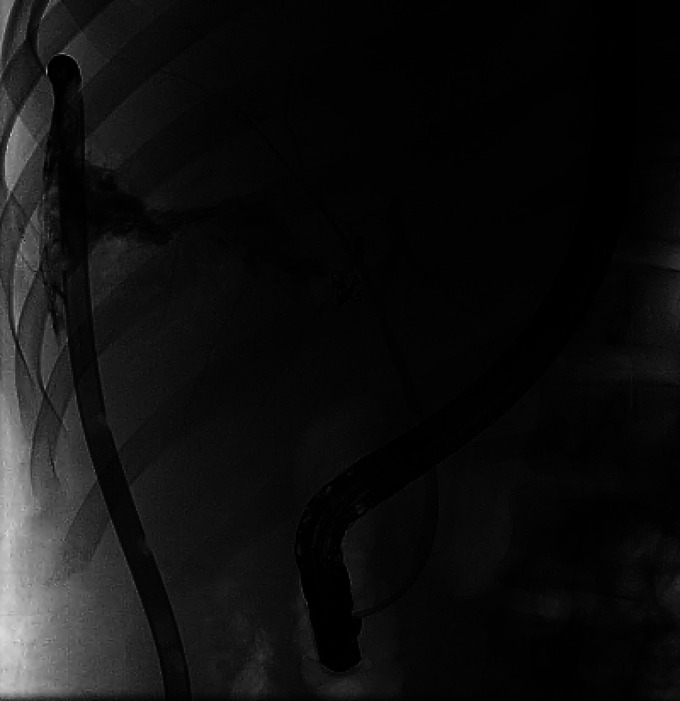
Index endoscopic retrograde cholangiopancreatography with cholangiogram with high-grade bile leak from the right hepatic duct just above the bifurcation.

**Figure 3.**
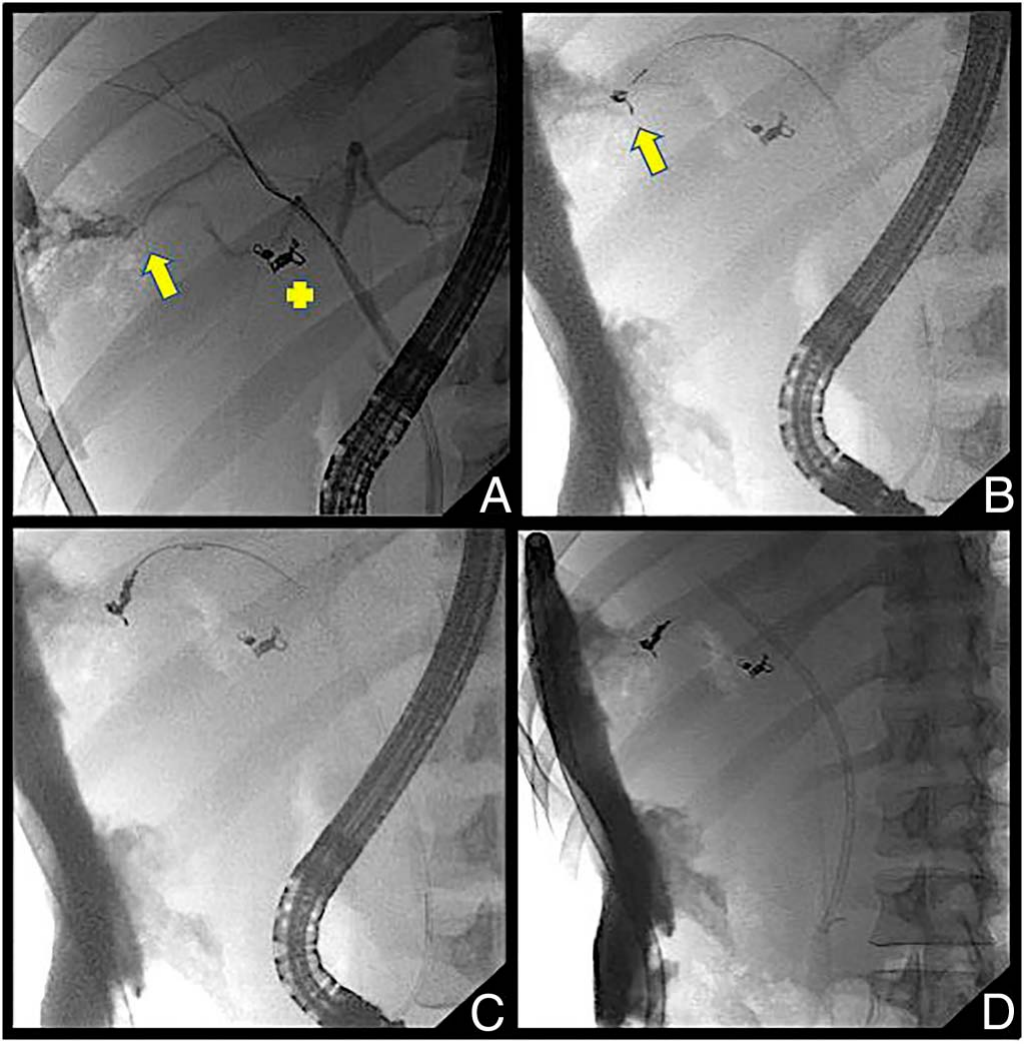
Sequential cholangiograms. (A) Bile leak from the right intrahepatic branch (yellow arrow), (B and C) placement of intraductal biliary coils at the bile leak site and (D) plastic biliary stent in place.

Endoscopic retrograde cholangiopancreatography (ERCP) was recommended. Cholangiograms noted early contrast extravasation concerning for a high-grade bile leak from the right hepatic duct (RHD) just above the hilar bifurcation, for which biliary sphincterotomy was performed followed by the placement of an 8.5 French × 12 cm plastic biliary stent bridging across the leak for preferential drainage (Figure 2). The patient initially improved; however, 3 days after the ERCP, there was increased bilious output (900 mL/d) from the percutaneous intrahepatic drain. Therefore, the patient underwent a second ERCP. After stent removal, cholangiograms noted improvement of the RHD bile leak; however, a second high-grade bile leak was noted off the RHD intrahepatic branches (Figure 3). The RHD intrahepatic bile leak was not noted on the index ERCP. However, this was thought to be secondary to the high-grade nature of the primary bile leak noted at the RHD just above the hilar bifurcation. Given the length of the plastic biliary stent placed at the index ERCP, this should have sufficed for preferential drainage for both leaks (RHD and RHD intrahepatic branch).

**Figure 4.**
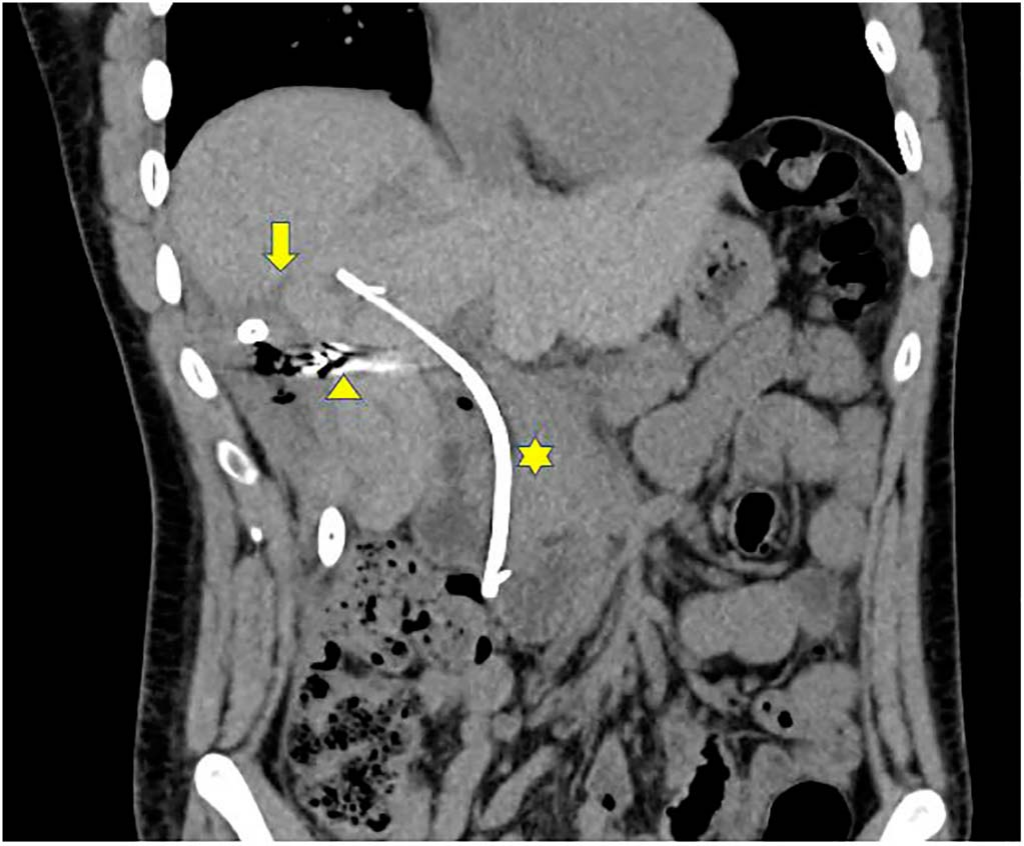
Six weeks after endobiliary coil embolization showing right lobe necrotic collection and intrahepatic drain (yellow arrow), endobiliary coils (yellow arrowhead), and the biliary stent (yellow star).

Owing to the peripheral location and refractory nature of the bile leak, biliary embolization was deemed necessary. Selective intrahepatic branch cannulation was performed with a long-wire sphincterotome (Cotton CannulaTome; Cook Medical) over an angle tipped 0.035 inches by a 450-cm guidewire (Jagwire; Boston Scientific). Sterile water was flushed through the sphincterotome after guidewire removal. Endobiliary coils (Tornado 0.035 inch, 5–3 mm; Cook Medical) were loaded and deployed by pushing them through the sphincterotome with the guidewire. A total of 3 endobiliary coils were deployed. The biliary stent was replaced (Figure 3). Bilious drain output markedly decreased. This improvement in bilious drain output was attributed to biliary coil embolization because the length of the plastic stent placed during the second ERCP was the same as the index procedure. Pain control and diet tolerance were achieved. He was discharged home with the right intrahepatic drain in position. Six weeks after biliary coil embolization, the patient continued to improve and reported low-volume drainage with improvement in the size of right lobe necrotic collection (Figure 4).

## DISCUSSION

Bile leaks are classified as low-grade or high-grade based on cholangiograms performed during ERCP, with high-grade leaks identified before intrahepatic opacification vs low-grade leaks which require complete or near-complete filling of intrahepatic ducts to be identified.^16,17^ Our patient had a refractory, high-grade intrahepatic leak, and the decision was made to embolize the duct with metallic coils. To the best of our knowledge, this is the third reported case of endobiliary coil placement alone, all of which lead to the resolution of a refractory biliary leak after initial failure at intervention.

For complex or refractory biliary leaks, innovative endoscopic modalities have been pursued. Kahaleh et al^11^ described a series of 16 patients who failed to respond to conventional endoscopic management of bile leaks who subsequently underwent the placement of a covered self-expandable metal stent with 15 patients with eventual bile leak resolution. The advantage of metal stents is thought to be their larger diameter and longer patency; however, migration of the stent is an important concern as was noted in 2 patients and the overall cost-effectiveness.

Injection of N-butyl-2-cyanoacrylate (CYA), a tissue glue monomer that solidifies on contact with body fluids at neutral pH, is another such novel method. Seewald et al^12^ described a series of 9 patients in which primary endoscopic treatment was unsuccessful who then underwent CYA occlusion during ERCP resulting in the successful resolution of bile leak in 7 patients. Schelhammer et al^13^ first described the successful embolization of a cystic duct stump after cholecystectomy. In addition, Brown et al^14^ reported the successful treatment of a persistent biliary leak from the cystic duct stump after cholecystectomy using percutaneous transabdominal access of the cystic duct and coil embolization. Kirtane et al^15^ reported the first successful intrahepatic coil embolization for persistent biliary leak after abdominal trauma. Although the use of endoscopic coil embolization has also been perfromed in conjunction with CYA,^16,18^ there has been at least 1 case report of cholangitis due to CYA and coil migration.^16^ Coil embolization has also been accomplished successfully through a percutaneous approach.^19,20^ At least 2 cases of complications because of coil migration with the percutaneous approach have been reported.^21,22^

Our case adds to limited data on the effective use of endobiliary coils for refractory bile leaks. This report was written with the intention of detailing a new method and instruments to be considered in cases of refractory bile leaks after initial interventions have failed, including sphincterotomy and biliary stent placement for preferential biliary drainage. In addition, patients should have close postprocedure monitoring, given the potential complications associated with this technique, such as coil migration and cholangitis.

## DISCLOSURES

Author contributions: D. Bhakta and T. DaVee wrote and approved the article. B. Dutra and M. Welborn reviewed the literature. All authors edited and revised the article for intellectual content. D. Bhakta is the article guarantor.

Financial disclosure: None to report.

Informed consent was obtained for this case report.
